# *MIR4435-2HG* as a possible novel predictive biomarker of chemotherapy response and death in pediatric B-cell ALL

**DOI:** 10.3389/fmolb.2024.1385140

**Published:** 2024-04-30

**Authors:** Yulieth Torres-Llanos, Jovanny Zabaleta, Nataly Cruz-Rodriguez, Sandra Quijano, Paula Carolina Guzmán, Iliana de los Reyes, Nathaly Poveda-Garavito, Ana Infante, Liliana Lopez-Kleine, Alba Lucía Combita

**Affiliations:** ^1^ Cancer Biology Group, Instituto Nacional de Cancerología, Bogotá, Colombia; ^2^ Department of Interdisciplinary Oncology, Louisiana State University Health Sciences Center, New Orleans, LA, United States; ^3^ Versiti Blood Research Institute, Milwaukee, WI, United States; ^4^ Department of Microbiology, Pontificia Universidad Javeriana, Bogotá, Colombia; ^5^ Department of Pediatrics, Hospital Militar Central, Bogotá, Colombia; ^6^ Department of Pediatrics, Hospital Universitario San Ignacio, Bogotá, Colombia; ^7^ Department of Statistics, Universidad Nacional de Colombia, Bogotá, Colombia; ^8^ Department of Microbiology, School of Medicine, Universidad Nacional de Colombia, Bogotá, Colombia

**Keywords:** B-cell acute lymphoblastic leukemia, biomarkers, MRD, gene expression, DNA methylation, prognosis, treatment response

## Abstract

**Introduction:** Although B-cell acute lymphoblastic leukemia (B-cell ALL) survival rates have improved in recent years, Hispanic children continue to have poorer survival rates. There are few tools available to identify at the time of diagnosis whether the patient will respond to induction therapy. Our goal was to identify predictive biomarkers of treatment response, which could also serve as prognostic biomarkers of death, by identifying methylated and differentially expressed genes between patients with positive minimal residual disease (MRD+) and negative minimal residual disease (MRD-).

**Methods:** DNA and RNA were extracted from tumor blasts separated by immunomagnetic columns. Illumina MethlationEPIC and mRNA sequencing assays were performed on 13 bone marrows from Hispanic children with B-cell ALL. Partek Flow was used for transcript mapping and quantification, followed by differential expression analysis using DEseq2. DNA methylation analyses were performed with Partek Genomic Suite and Genome Studio. Gene expression and differential methylation were compared between patients with MRD−/− and MRD^+/+^ at the end of induction chemotherapy. Overexpressed and hypomethylated genes were selected and validated by RT-qPCR in samples of an independent validation cohort. The predictive ability of the genes was assessed by logistic regression. Survival and Cox regression analyses were performed to determine the association of genes with death.

**Results:** *DAPK1*, *BOC*, *CNKSR3*, *MIR4435-2HG*, *CTHRC1*, *NPDC1*, *SLC45A3*, *ITGA6*, and *ASCL2* were overexpressed and hypomethylated in MRD^+/+^ patients. Overexpression was also validated by RT-qPCR. *DAPK1*, *BOC*, *ASCL2*, and *CNKSR3* can predict refractoriness, but *MIR4435-2HG* is the best predictor. Additionally, higher expression of *MIR4435-2HG* increases the probability of non-response, death, and the risk of death. Finally, *MIR4435-2HG* overexpression, together with MRD+, are associated with poorer survival, and together with overexpression of *DAPK1* and *ASCL2*, it could improve the risk classification of patients with normal karyotype.

**Conclusion:** *MIR4435-2HG* is a potential predictive biomarker of treatment response and death in children with B-cell ALL.

## 1 Introduction

B-cell acute lymphoblastic leukemias (B-cell ALL) are the most frequent neoplasms in children (Pui et al., 2008). Cure rates for acute lymphoblastic leukemias (ALL) have improved remarkably in the last 4 decades; however, while developed countries achieve 80% cure rates, those rates are around 60% in developing countries (Vera et al., 2012). Some studies have shown that, even under the same treatment protocols, Hispanic children have worse survival and treatment response compared to White and Asian children (Matasar et al., 2006; Walsh et al., 2013; Walsh et al., 2014). The mechanisms underlying these differences in survival rates are still unknown.

Currently, clinical parameters such as leukocyte count, age, extramedullary infiltration, chromosomal translocations, and minimal/measurable residual disease (MRD) classify patients into risk groups. MRD is the most used variable to define treatment response (van Dongen Jacques et al., 1998; Van Dongen JJM et al., 2015). However, due to low survival rates in our patients, it is possible to propose that those variables do not fully define risk groups, which leads to incorrect selection of chemotherapy protocol, affecting patient survival (Sok et al., 2022).

In ALL, gene expression alterations not only result from mutations; alterations at the epigenetic level also play a relevant role in this pathology (Garcia-Manero et al., 2009; Newton et al., 2014; Hu and Shilatifard, 2016; Nordlund and Syvänen, 2018). Thus, epigenetic alterations, including aberrant DNA methylation, could act as important molecular mechanisms in developing resistance to treatment of ALL (Newton et al., 2014). In bone marrow (BM), DNA methylation patterns change during normal hematopoiesis and play an essential role in lineage differentiation (Cullen et al., 2014; Wainwright and Scaffidi, 2017). As in normal cells, tumor cells may also depend on specific DNA methylation patterns to acquire their phenotype and maturation patterns (Patel and Vanharanta, 2017; Wainwright and Scaffidi, 2017; Poli et al., 2018). Therefore, the characterization of aberrant patterns in DNA methylation in tumors can provide important clues about how gene expression is regulated in these pathologies (Nordlund and Syvänen, 2018). Hogan et al., 2015 found that patients with relapses presented promoter hypermethylation and identified a clear signature of differentially expressed genes at the time of diagnosis and relapse; moreover, this signature differs between early-relapse patients and to late-relapse patients. Similarly, aberrant promoter methylation has been associated with MRD. For example, aberrant methylation of the promoters of the *RASSF6* and *RASSF10* genes has been observed in adults with B-cell ALL, which can be detected in peripheral blood and could be useful as potential biomarkers to measure MRD (Younesian et al., 2019). Furthermore, it has been reported that promoter methylation of the *TLX3* and *FOXE3* genes in children with B-cell ALL differentiates MRD + patients from MRD-patients (Chatterton et al., 2014).

Although differential methylation and gene expression patterns have been observed between samples at diagnosis and in relapse, whether these variables could be tools to predict treatment response, including relapse or death, is yet to be determined. Also, a CpG island methylation analysis identified candidate genes as biomarkers of pediatric ALL subgroups and their correlation with disease prognosis (Stumpel et al., 2009). Identifying genomic markers, derived from methylation and gene expression analysis, could improve risk classification, and define patient prognosis.

We hypothesized that gene expression and DNA methylation of blasts obtained at diagnosis differ between MRD+ and MRD-patients and that by comparing these two conditions, candidate genes predictive of treatment response and death could be identified. We collected BM samples obtained at diagnosis, purified leukemic blasts, and compared gene expression and DNA methylation profiles between MRD+ and MRD-patients at the end of induction, looking for overexpressed and hypomethylated genes in MRD + patients. Subsequently, we evaluated if the selected genes could predict response to induction chemotherapy, or death. The search for new genomic biomarkers will improve risk classification and, in the future, patient survival.

## 2 Materials and methods

### 2.1 Patient samples

Forty-three patients with B-ALL who attended the Instituto Nacional de Cancerología, Hospital Militar Central and Hospital Universitario San Ignacio (Bogotá, Colombia) between 2017 and 2021 were included. The discovery cohort consisted of 13 BM samples taken at the time of the diagnosis in which RNA-seq/DNA methylation protocols were performed. Sequencing data from 12 patients was used to enrich the survival analyses. Eighteen BM samples taken at the time of the diagnosis were included in the validation cohort by RT-qPCR.

Newly diagnosed patients were included in the study when they entered to the institutions for symptomatology associated with ALL and after verification of the inclusion criteria (not having received chemotherapy, not having another type of cancer, not having genetic diseases and being younger than 18 years old). The diagnosis was confirmed using flow cytometry (Van Dongen JJM et al., 2012) and morphological analysis of BM. This study was conducted following the recommendations of the Colombian Regulation for Research in Humans (Resolution 8430 of 1993, Ministry of Health of Colombia) and in accordance with the Declaration of Helsinki and approved by each participating institution’s Institutional Review Boards (IRB). All methods for nucleic acid analysis were approved by the LSUHSC Translational Genomics Core’s Institutional Biosafety Committee protocol number 17370. Informed consent was signed by the parents of all participants. Each patient was treated according to the assigned risk and in accordance with the Berlin-Frankfurt-Munich protocol (Stary et al., 2014). Patients with treatment abandonment or non-adherence to it were excluded.

According to the Berlin-Frankfurt-Munich protocol, response to induction therapy was evaluated by flow cytometry detecting MRD at day 15, where patients with <0.1% residual blasts in BM were MRD-, and patients with >0.1% residual blasts were considered MRD+. At day 33, patients with <0.01% residual blasts in BM were MRD-, while patients with >0.01% residual blasts were MRD+ (Stary et al., 2014). Therefore, we considered patients with MRD-day15 and MRD-day33 as MRD−/− and patients with MRD + day15 and MRD + day33 as refractory patients or MRD^+/+^.

### 2.2 Blasts isolation and purification

BM samples were collected by a hemato-oncologist and processed within 24 h after sample collection. First, mononuclear cells were separated from BM by density-gradient centrifugation (Lymphoprep, Lonza). The blasts were separated using magnetic microbeads coated with anti-CD19 or anti-CD34 antibodies, followed by MACS column enrichment (Miltenyi, Bergisch Gladbach, Germany). The purity of sorted blasts was assessed with CD34-PERCPCy5.5, CD45-V500, CD19-PECy7, and CD10 APC antibodies. Data was acquired in a FACSCanto II flow cytometer (Becton/Dickinson Biosciences, San Jose, CA), using the FACSDiva software program. Infinicyt software (Cytognos SL, Salamanca, Spain) was used for data analysis (Cruz-Rodriguez et al., 2016).

### 2.3 DNA and RNA extraction

DNA and RNA were extracted from MACS-sorted blasts using the Allprep mini kit and the robotic workstation QIAcube (Qiagen, Hilden, Germany). RNA quality was evaluated using the Agilent RNA 6000 Nano and Pico kits in the Agilent 2100 Bioanalyzer. RNA concentration was calculated using the Qubit™ RNA High Sensitivity and Broad Range kits, while DNA concentration was calculated using the Qubit dsDNA HS Assay Kit (Thermo Fisher Scientific).

### 2.4 Library preparation and RNA sequencing

Samples with RIN >6 and purity by flow cytometry with >90% blasts were selected for RNA-seq. For RNA library preparation, 300 ng of total RNA was used. TruSeq Stranded mRNA RNA libraries were prepared following Illumina’s protocol. Resulting libraries were sequenced at 2 × 75 bp on a NextSeq550 sequencer system at the Stanley S. Scott Cancer Center’s Translational Genomics Core at LSUHSC-New Orleans. On average, more than 50 million reads per sample were obtained. FASTQ files were uploaded to Partek Flow for analysis. First, removal of contaminant sequences (rDNA, mtrDNA, tRNA) was done with Bowtie 2.0 v2.2.5. Reads were aligned to the hg38 version of the human genome, using STAR 2.7.3a. Genes were quantified with RefSeq 96. For the analysis, genes with less than 5 reads in at least 80% of the samples were excluded. One sample with a low correlation (<0.4) with respect to the others was removed. Normalization was done with the Median Ratio and differential expression analysis was assessed with DESEQ2. Hierarchical clustering, pathways (KEEG) and GO terms were all analyzed in Partek Flow.

### 2.5 DNA methylation assay

Bisulfite conversion was performed in 500 ng of DNA for each sample following the recommendations of the EZ DNA Methylation-Startup Kit (Catalogue number D5001, Zymo Research, United States). Bisulfite-converted DNA was amplified and hybridized to the Infinium Methylation EPIC Kit chips and scanned on the Illumina’s iScan. Analysis of the methylation assays was done in Partek Genomic Suite. Low-confidence probes (*p*-value >0.05) and probes mapped to X and Y chromosomes were excluded. Normalization was done using NOOB (normalization for Illumina Infinium methylation arrays).

### 2.6 RT-qPCR

Total RNA from sorted blasts was treated with DNase I Amplification Grade (Invitrogen, United States) prior to reverse transcription. cDNA was synthesized using the SuperScript III First-Strand Synthesis SuperMix Kit (Invitrogen, United States), following the manufacturer’s procedures. TaqMan probes were used to quantify mRNA expression levels of candidate genes obtained by RNA-seq analysis (Assay IDs: *DAPK1* Hs00234489_m1; *NPDC1* Hs00209870_m1; *CNKSR3* Hs00295109_m1; *SLC18A2* Hs00996835_m1; *CTHRC1* Hs00298917_m1; *BOC* Hs00264408_m1; *SLC45A3* Hs00263832_m1; *GAPDH* Hs99999905_m1, *ASCL2* Hs00270888_s1; *MIR4435*-*2HG* Hs03680374_m1). The reaction was amplified in a QuantStudio 12 K plex Real-Time PCR machine (Applied Biosystems). The 2^−ΔΔCT^ method was used to estimate the fold induction of each gene using *GAPDH* and Ct values to determine the fold change (FC) for each sample. A pool of samples was used as internal calibrator, as well as water as negative control. Assays were done in duplicate.

### 2.7 Statistical analysis

#### 2.7.1 Transcriptomic and methylation data analysis

Normalization and differential expression analysis were performed using the Deseq2 library in RStudio. Differentially expressed genes (DEGs) were selected if they had a *p*-value <0.05 and a FC > 2. ggplot library was used to generate heatmaps, and GenomeStudio to calculate beta values for each hybridized probe. The Partek Genomic Suite was used for differential methylation analysis to identify genes with differentially methylated CpGs (GDMCpGs). GDMCpGs were chosen if they had FC > 2 and FDR <0.05. Enrichment analysis and functional gene annotation were performed Clusterprofiler in RStudio. Pearson correlation was used to determine correlation between overexpressed genes and hypomethylated probes; those with an inverse correlation less than −0.50 and a *p*-value <0.05 were selected.

#### 2.7.2 Experimental design

**FIGURE 1:**
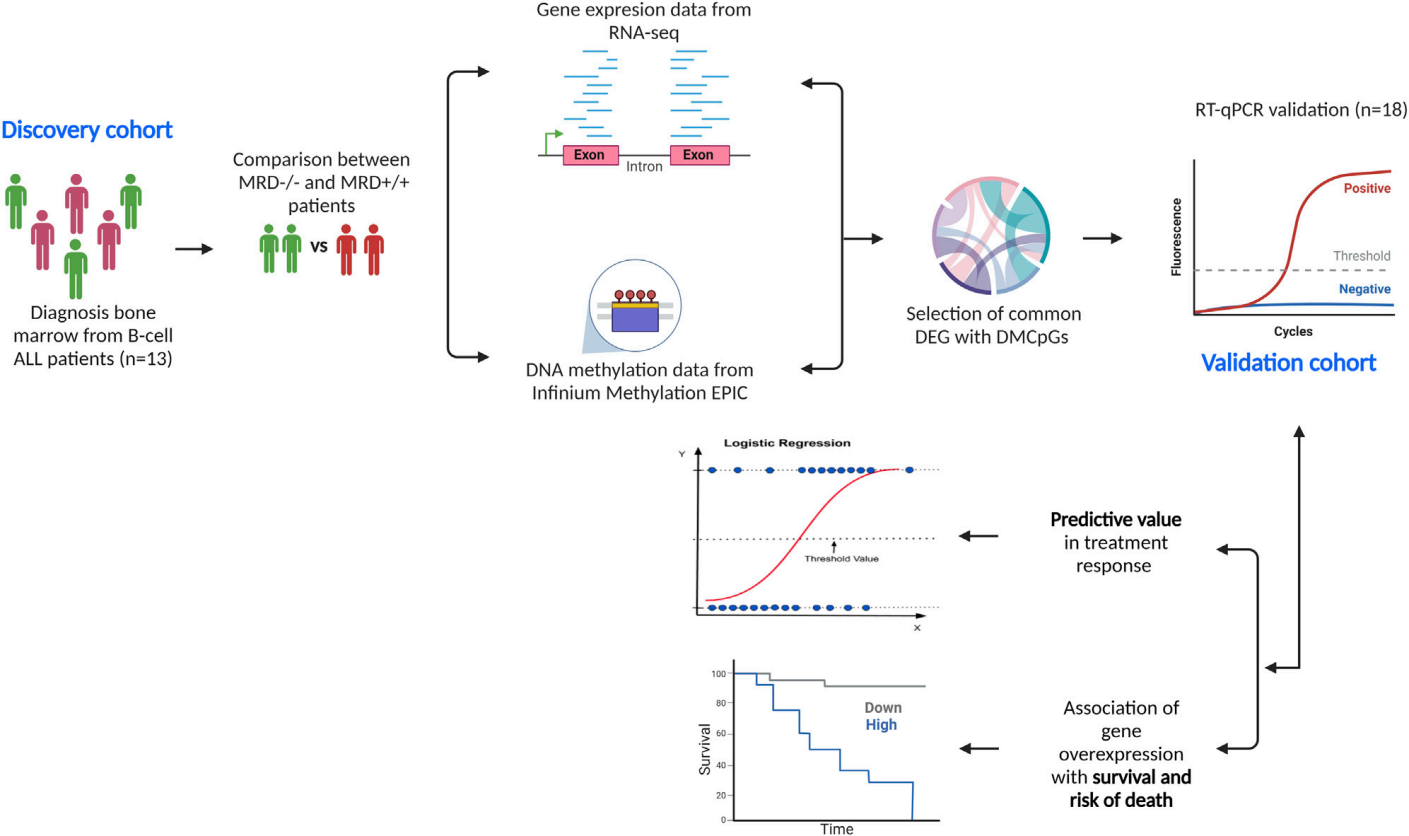
Methodological design of the study. We describe the descriptive cohort (*n* = 13) in which DNA methylation and gene expression analyses were performed separately, comparing the results between MRD−/− vs. MRD^+/+^. From the comparisons, 10 DEGs with differentially methylated CpGs were selected and verified in the validation cohort (*n* = 18) via RT-qPCR. Subsequently, the predictive ability of genes on outcomes such as achievement of complete remission at end of induction and death was tested. The relationship between gene overexpression and overall survival and risk of death was also assessed.

Figure 1 describes the methodological design of the study. Gene and methylation profiles of induction treatment were compared between MRD−/− vs. MRD^+/+^. Treatment response was the only variable used to define profiles in each comparison.

#### 2.7.3 RT-qPCR analysis

Spearman correlation was used to determine any correlation between normalized RNA-seq counts and FC values for RT-qPCR. Genes with *p*-value <0.05 and r > 0.72 were selected. Mann-Whitney test was used to compare FC between MRD−/− and MRD^+/+^ patients. GraphPad software was used for statistical tests and graphic images. In both analyses, outliers were identified by the ROUT method (Q = 1%) and excluded from the analyses.

#### 2.7.4 Clinical data analysis

To compare clinical variables between patient cohort, *t*-test and chi-square tests were used. The follow-up time for relapse and death was 2 years. Logistic regression analysis was performed to evaluate whether candidate genes could predict treatment response. Survival analyses were estimated according to gene expression using Kaplan-Meier curves. Cox regression was used to determine whether gene expression conferred a higher risk of death. The Youden index of normalized RNA-seq counts was used to define the cutoff threshold for overexpression for each gene.

## 3 Results

Table 1 describes the clinicopathological characteristics of the patients included in the discovery cohort, and Supplementary Material S1 shows the clinical variables of the validation cohort. As can be observed, no differences in clinical variables between MRD^+/+^ and MRD−/− patients, except for risk, were found. However, this was to be expected because MRD^+/+^ patients are considered intermediate to high risk, whereas MRD−/− patients may be low to intermediate risk. Interestingly, more than seventy percent of patients had normal karyotype. The MRD−/− group had 1 death related to relapse and progression and another one due to febrile neutropenia. Similarly, the MRD^+/+^ group had one death due to relapse and progression and 4 deaths during the induction phase (very aggressive disease).

**TABLE 1:** Clinical characteristics of the DISCOVERY cohort.

Clinical characteristics	MRD^+/+^ (*n* = 6)		MRD−/− (*n* = 7)		
	n (%)	Mean (range)	n (%)	Mean (range)	*p*-value
**AGE (years)**		11 (3–17)		10.7 (3–15)	0.91
**SEX**					
Female	2 (33.3)		2 (28.5)		0.85
Male	4 (66.7)		5 (71.5)		
WBC (cel/mL)		90.7 (9.7–292)		35.99 (6.22–91.8)	0.22
**RISK**					
Low	0 (0)		1 (14.2)		0.03
Intermediate	2 (33.3)		6 (85.8)		
High	4 (66.7)		0 (0)		
**EXTRAMEDULLAR INFILTRATION**					
Yes	1 (16.6)		2 (28.5)		0.61
No	5 (83.4)		5 (71.5)		
**CORTICOID RESPONSE**					
Yes	5 (83.4)		7 (100)		0.26
No	1 (16.6)		0 (0)		
**CARIOTYPE/MOLECULAR ALTERATIONS**					
Normal	4 (66.8)		5 (71.4)		0.56
t (1; 19)	1 (16.6)		1 (14.3)		
t (9; 22)	1 (16.6)		0 (0)		
t (3; 14)	0 (0)		1 (14.3)		
**RELAPSE**					
Yes	1 (16.6)		1 (14.2)		0.90
No	5 (83.4)		6 (85.8)		
**DEATH**					
Yes	4 (66.7)		2 (28.5)		0.16
No	2 (33.3)		5 (71.5)		

MRD^+/+^+/+ indicates minimal residual disease positive. MRD−/−-/- indicates minimal residual disease negative.

### 3.1 Identification of DEGs

**FIGURE 2:**
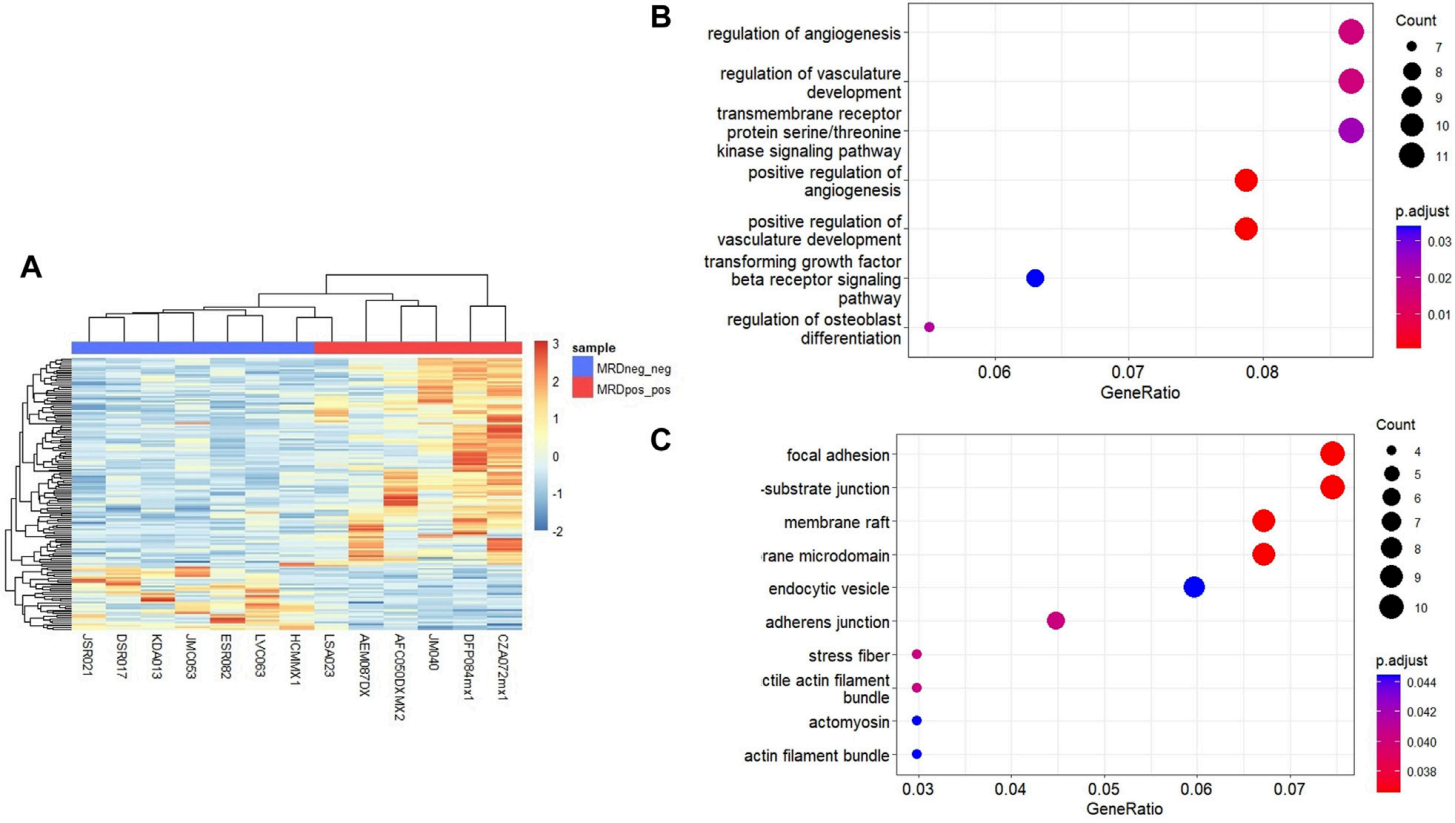
Differentially expressed genes between MRD+ and MRD-patients. **(A)** Heatmap of DEGs between MRD−/− and MRD^+/+^. Each column represents an individual patient and horizontal axis indicates each differentially expressed gene. In blue scale downregulated genes, and the red scale shows upregulated genes (FC > 2 and <−2, *p*-value <0.05). **(B)** Dot plot showing the 7 most significant biological processes in which differentially expressed genes between conditions are grouped. **(C)** Dot plot showing the 10 most significant cellular components in which differentially expressed genes between conditions are grouped.

To identify genes that could differentiate MRD^+/+^ patients from MRD−/− patients, we performed RNA-seq and MethylationEPIC in nucleic acids extracted from immunomagnetic column-enriched leukemic blasts obtained at the time of diagnosis. MRD status was obtained from medical charts at day 15 and 33. We then compared the gene expression and DNA methylation profiles between MRD−/− vs. MRD^+/+^ patients. Unsupervised hierarchical cluster analysis showed 117 upregulated and 36 downregulated DEGs MRD^+/+^ vs. MRD−/− patients (Figure 2A). Among the biological processes with the highest number of genes involved are neutrophil activation, serine/threonine membrane receptors, extracellular matrix organization, among others ((Figure 2B). The cellular components with the highest number of genes involved include cell-cell junction, adhesion, vesicles, among others ((Figure 2C).

### 3.2 Identification of GDMCpGs

**FIGURE 3:**
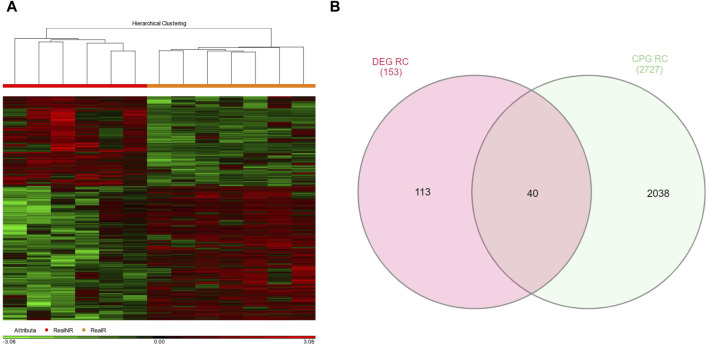
Genes with differentially methylated CpGs between MRD+ and MRD-patients. **(A)** Heatmap of differentially methylated GDMCpGs between MRD−/− and MRD^+/+^. Each column represents an individual patient and horizontal axis indicates the beta values of each differentially methylated CpG. Hypomethylated probes are shown in green and hypermethylated probes in red. The MRD−/− patient group is shown in orange and the MRD^+/+^ patient group in red. FC > 2 and <−2, *p*-value <0.05. **(B)** Venn diagram identifying common DEGs and GDMCpGs after MRD−/− vs. MRD^+/+^ comparison.

Additionally, a total of 2726 GDMCpGs were identified between MRD^+/+^ and MRD−/− patients (Figure 3A). To establish a correlation between DEGs and their corresponding methylation levels, we compared DEGs and GDMCpGs to determine if there were common genes between the two techniques. This comparison revealed 40 common genes between MRD +/+ and MRD −/− patients (Figure 3B). Notably, we observed a significative inverse correlation involving the overexpression of 10 genes and their associated CpGs hypomethylation (Table 2).

**TABLE 2:** Relationship between beta values of CPGS sites and normalized RNASEQ counts of differentially expressed genes between MRD^+/+^ and MRD−/− patients.

	Relation to CpGs island	AFC050	AEM087	LSA023	JM040	CZA072	DFP084	KDA013	DSR017	JSR021	HCMMX1	JMC053	LVC063	ESR082	*p*-value	Rho
*CNKSR3*																
Normalized RNAseq counts		5.39	4.49	7.01	8.20	11.94	8.41	4.20	4.28	4.58	5.90	4.77	6.10	3.63	0.04	−0.57
cg00460149		0.10	0.22	0.07	0.06	0.09	0.09	0.24	0.60	0.13	0.21	0.20	0.07	0.48		
***CTHRC1***																
Normalized RNAseq counts		6.00	6.17	3.63	10.76	11.57	10.42	4.29	4.09	4.24	4.35	7.22	5.33	4.28	0,001	−0.78
cg01224234		0.47	0.47	0.22	0.05	0.06	0.17	0.79	0.83	0.81	0.82	0.07	0.73	0.83		
***NPDC1***																
Normalized RNAseq counts		11.79	11.51	5.83	7.91	13.17	12.45	4.66	5.68	5.32	4.71	6.42	8.90	9.16	0,003	−0.74
cg14190761	Island	0.07	0.15	0.20	0.09	0.05	0.08	0.29	0.24	0.18	0.18	0.20	0.26	0.18		
***DAPK1***																
Normalized RNAseq counts		13.20	10.94	5.75	12.47	12.19	13.07	10.23	4.65	7.59	9.04	4.98	8.69	5.37		
cg08719486	N_Shore	0.74	0.43	0.55	0.43	0.14	0.35	0.82	0.72	0.88	0.47	0.82	0.85	0.47	0.14	−0.42
cg11518830		0.07	0.13	0.35	0.07	0.17	0.10	0.13	0.52	0.44	0.16	0.55	0.18	0.75	0,0001	−0.87
***SLC45A3***																
Normalized RNAseq counts		8.83	8.50	6.99	9.67	12.74	12.34	7.11	5.95	6.40	5.27	9.32	7.30	5.46		
cg01455178	N_Shore	0.32	0.72	0.52	0.71	0.15	0.24	0.74	0.71	0.75	0.80	0.78	0.81	0.39	0.02	−0.60
cg04896348		0.37	0.69	0.63	0.09	0.15	0.41	0.61	0.58	0.62	0.61	0.61	0.67	0.65	0,007	−0.69
***ITGA6***																
Normalized RNAseq counts		14.90	10.68	8.75	10.51	15.81	15.34	10.62	11.99	8.73	10.83	10.01	10.64	8.06		
cg07592198		0.12	0.46	0.46	0.82	0.29	0.36	0.73	0.80	0.70	0.55	0.75	0.75	0.74	0,008	−0.69
cg13586889		0.05	0.14	0.08	0.08	0.05	0.06	0.39	0.19	0.37	0.16	0.22	0.10	0.20	0.05	−0.53
***MIR4435-2HG***																
Normalized RNAseq counts		6.30	5.54	4.90	6.04	9.43	7.20	4.55	4.74	4.89	4.55	5.15	4.44	4.42	0.04	−0.56
cg24783876		0.08	0.69	0.53	0.08	0.26	0.06	0.22	0.68	0.73	0.79	0.65	0.42	0.67		
***SLC18A2***																
Normalized RNAseq counts		4.08	4.3	4.85	6.1	9.46	11.4	4.29	4.28	4.48	4.05	4.25	4	3.63		
cg03570973	N_Shore	0.81	0.5	0.4	0.15	0.67	0.07	0.79	0.59	0.79	0.71	0.82	0.81	0.8	0.01	−0.63
cg08521987	Island	0.51	0.11	0.2	0.22	0.1	0.07	0.67	0.4	0.5	0.26	0.44	0.48	0.78	0.01	−0.64
cg27186877		0.12	0.1	0.08	0.09	0.08	0,007	0.17	0.17	0.16	0.06	0.54	0.34	0.36	0.09	−0.47
cg00512279	Island	0.63	0.15	0.19	0.18	0.22	0,006	0.7	0.47	0.47	0.2	0.59	0.53	0.74	0.01	−0.63
***ASCL2***																
Normalized RNAseq counts		5.52	7.08	4.19	7.8	8.71	8.15	5.18	4.09	4.37	3.63	3.63	4.36	4.27		
cg11644479	Island	0.74	0.57	0.23	0.30	0.47	0.44	0.75	0.74	0.73	0.84	0.78	0.72	0.74	0.03	−0.59
cg13762320	Island	0.91	0.74	0.61	0.60	0.68	0.71	0.93	0.77	0.83	0.86	0.94	0.91	0.91	0.03	−0.59
cg19284039	Island	0.83	0.62	0.29	0.36	0.56	0.45	0.85	0.50	0.87	0.89	0.85	0.78	0.72	0.09	−0.48
cg26051413	Island	0.74	0.62	0.32	0.30	0.54	0.43	0.69	0.46	0.82	0.65	0.79	0.73	0.69	0.12	−0.45
cg13930892	Island	0.61	0.23	0.43	0.17	0.21	0.23	0.55	0.32	0.42	0.28	0.74	0.78	0.74	0.01	−0.63
***BOC***																
Normalized RNAseq counts		4.52	6.09	4.50	4.59	8.17	7.60	4.10	4.09	4.06	3.63	5.04	4.52	4.27		
cg22413784		0.55	0.75	0.76	0.12	0.42	0.41	0.75	0.74	0.78	0.81	0.74	0.76	0.80	0,008	−0.70

### 3.3 Gene verification by RT-qPCR

**FIGURE 4:**
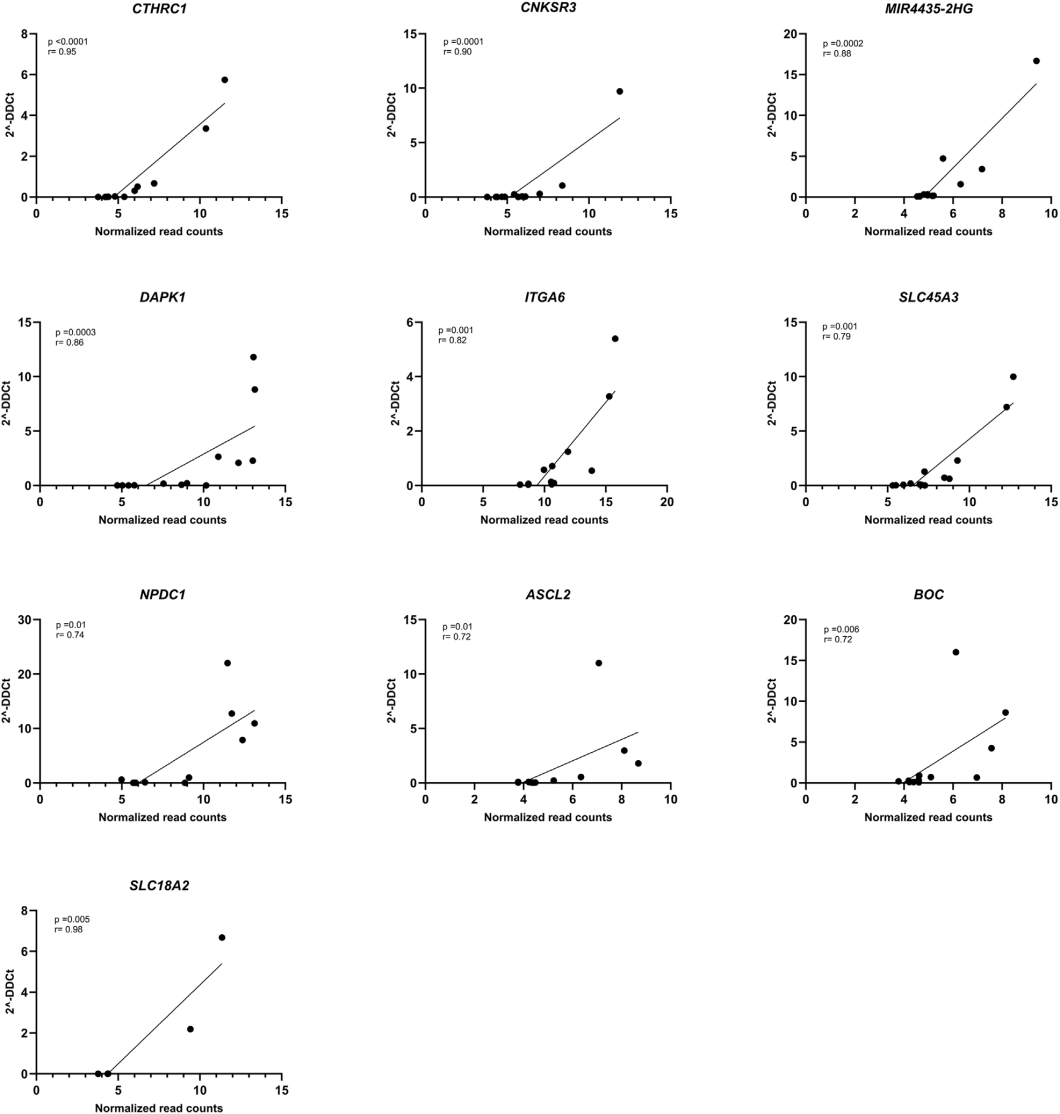
Correlation of RNA-seq data with RT-qPCR. Spearman correlation plot between normalized RNA-seq counts (*X*-axis) and 2^−ΔΔCT^ (*Y*-axis) of *DAPK1* (*n* = 13), *CNKSR3* (*n* = 12), *MIR4435-HG2* (*n* = 13), *CTHRC1* (*n* = 12), *NPDC1* (*n* = 10), *SLC45A3* (*n* = 13), *ITGA6* (*n* = 12), *ASCL2* (*n* = 11), *BOC* (*n* = 13), and *SLC18A2* (*n* = 6) genes. *p*-value <0.05 and r < 0.70.

Subsequently, gene expression was verified by RT-qPCR and all genes showed correlation between the normalized read counts (RNA-seq) and the 2^−ΔΔCT^ values obtained from RT-qPCR. Remarkably, *CTHRC1*, *CNKSR3*, *MIR4435-2HG*, *DAPK1*, and *ITGA6* demonstrated correlations exceeding 0.80 (Figure 4). Although *SLC18A2* was the gene with the best concordance, it was excluded from the analysis because only 6 patients were used for this analysis.

**FIGURE 5:**
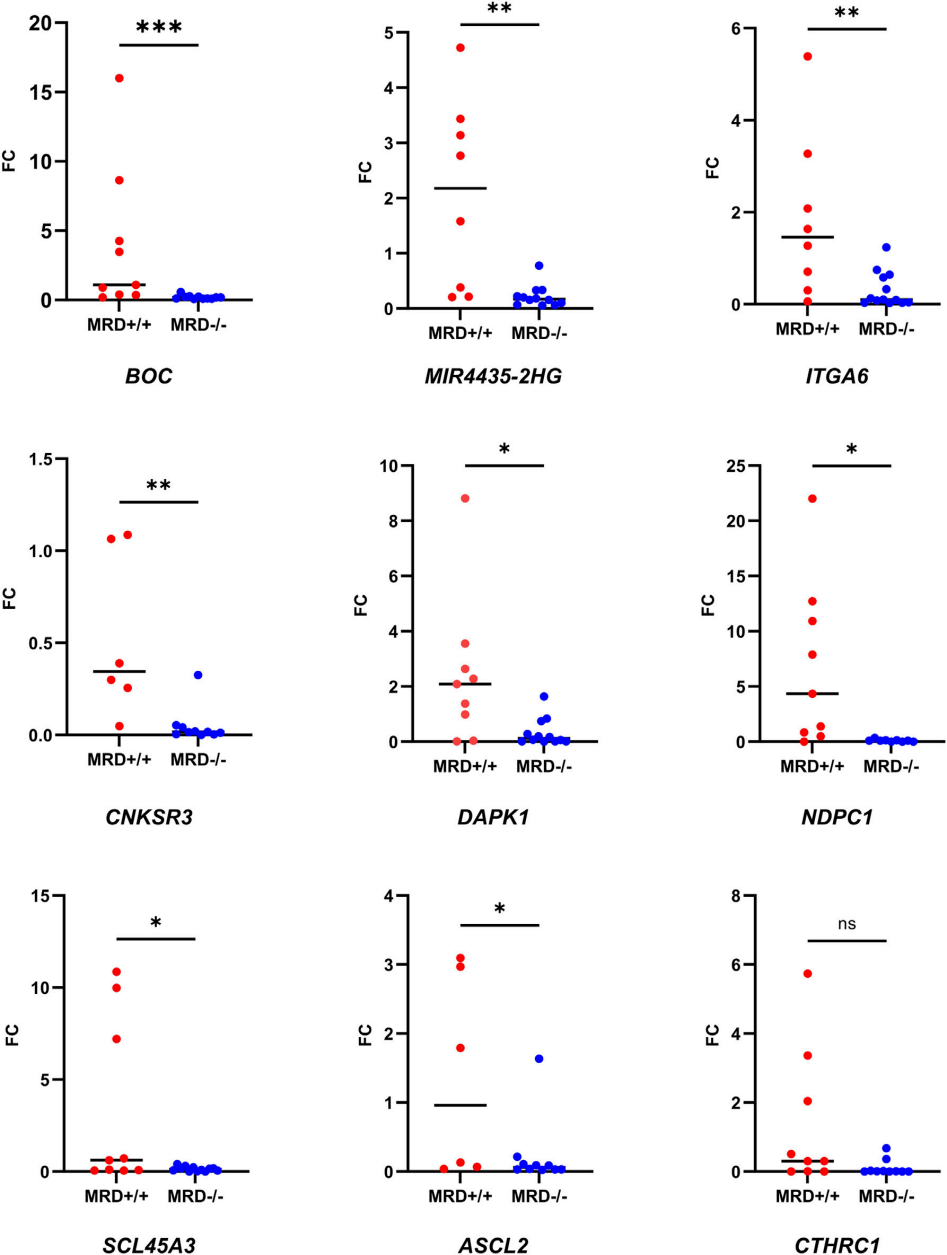
Comparison of fold change of the genes between MRD- and MRD + by RT-qPCR. Dot plot showing the comparison of FC of the nine selected genes between MRD−/− and MRD^+/+^ patients. Mann-Whitney test was used to compare FC between the groups (*p*-value = 0.03 (*), 0.0021 (**); 0.0008 (***), ns = >0.05)). Number of samples corresponding to each analysis: *MIR4435-HG2* (MRD−/− = 12 vs. MRD^+/+^ = 8), *DAPK1* (MRD−/− = 12 vs. MRD^+/+^ = 9), *CNKSR3* (MRD−/− = 10 vs. MRD^+/+^ = 6), *CTHRC1* (MRD−/− = 12 vs. MRD^+/+^ = 9), *NPDC1* (MRD−/− = 9 vs. MRD^+/+^ = 9), *SLC45A3* (MRD−/− = 12 vs. MRD^+/+^ = 9), *ITGA6* (MRD−/− = 13 vs. MRD^+/+^ = 8), *ASCL2* (MRD−/− = 10 vs. MRD^+/+^ = 6) and *BOC* (MRD−/− = 11 vs. MRD^+/+^ = 9).

Then, we wanted to test if the expression of these genes remained differential between MRD- and MRD + patients in the validation cohort. Due to the low incidence of the disease (Katz et al., 2015) and the small number of MRD + patients, samples from the MDR + patients in the discovery cohort were pooled with those from the validation cohort for RT-qPCR analyses. Consistent with RNA-seq results, all genes except *CTHRC1* were overexpressed in MRD^+/+^ patients by RT-PCR (Figure 5).

### 3.4 Predictive value of genes

**FIGURE 6:**
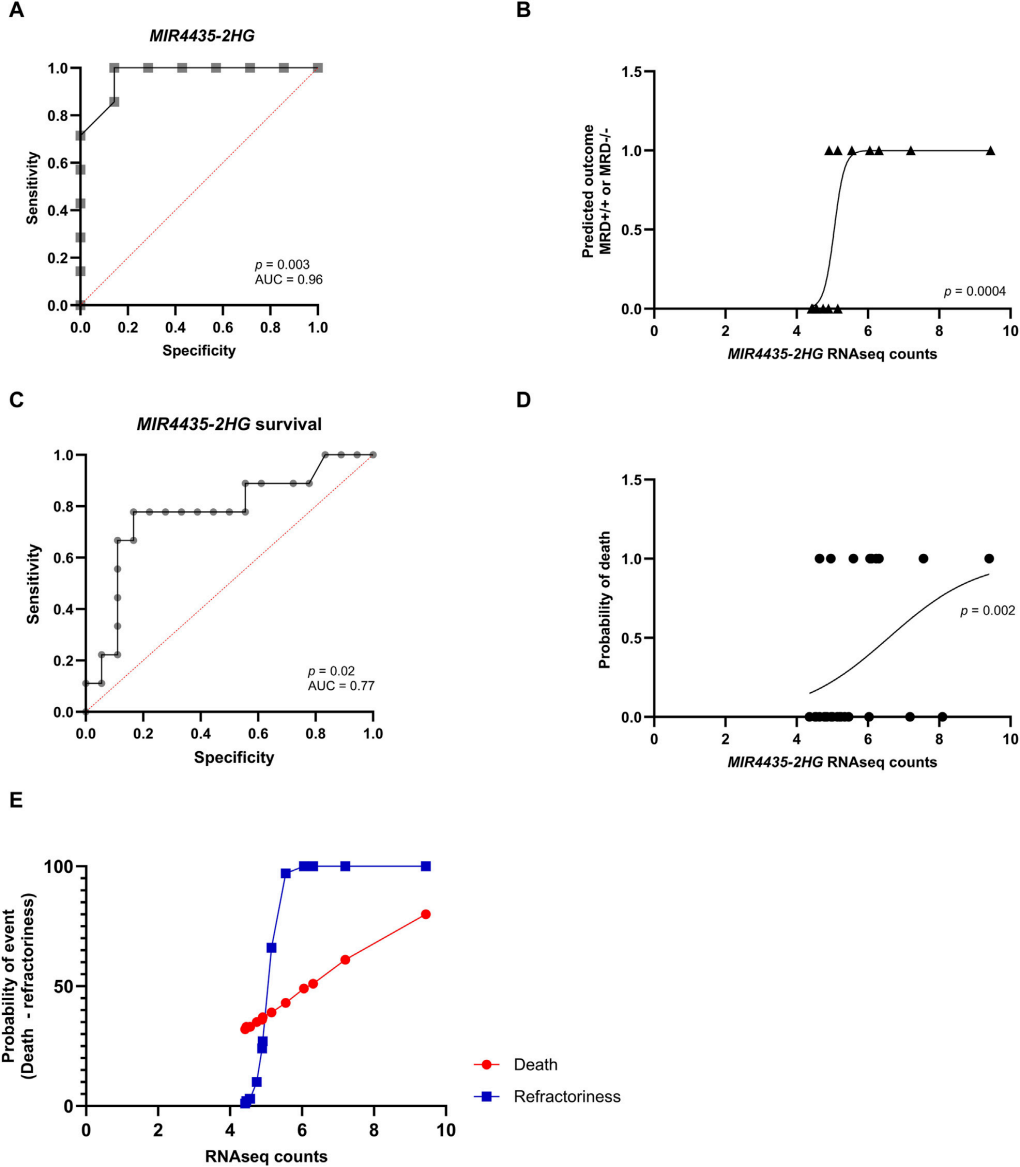
ROC curves and logistic regression graphs of *MIR4435-2HG* as a predictor of chemotherapy response and survival. Logistic regression analysis and ROC curves to determine the predictive capacity of the *MIR4435-2HG* and to determine its sensitivity and specificity. In logistic regressions, the probability of having one of the outcomes is represented on the *Y*-axis by numbers between 0 and 1, where 1 indicates that the patient does not respond to treatment and 0 indicates that the patient responds to treatment. On the *X*-axis, RNA-seq counts for the *MIR4435-2HG* gene are observed. **(A,B)** Prediction of if patient will be MRD−/− or MRD^+/+^ and **(C,D)** whether they will survive. Scatter plot of the relationship of RNAseq counts to the probability of death (red) or being refractory (blue) **(E)**. Likelihood ratio test, *p*-value <0.05 and area under the ROC curve >0.77 and *p*-value <0.05.

Afterward, we used logistic regression to evaluate whether genes could predict response to induction chemotherapy. Remarkably, *MIR4435-2HG* was found to be the best predictor of whether a patient would be MRD−/−, MRD^+/+^ (Figures 6A,B). It was observed that genes *DAPK1*, *BOC*, *ASCL2*, and *CNKSR3* could also predict whether the patient would be MRD−/− or MRD^+/+^ (Supplementary Material S2). To assess whether *MIR4435-2HG* could predict the risk of death, we performed logistic regression using our normalized read counts. We observed that *MIR4435-2HG* can predict death with good sensitivity and specificity (Figures 6C,D). Interestingly, we found that patients with counts >5.1 had a 66% probability of being MRD^+/+^ to treatment (refractory), and this probability increased proportionally to increases in gene expression. Similarly, the probability of death increased when counts were >7.0 (Figure 6E).

### 3.5 Relationship between gene expression and risk of death

With the aim of testing the potential of the identified genes as predictive biomarkers of mortality, four Cox regression models were performed to determine the clinical variables that influence patient survival (white blood cell count at diagnosis, age, extramedullary infiltration, response on day 8 of corticosteroid treatment, MRD on day 15, and at the end of treatment). induction) and overexpression of the identified genes.

The initial model incorporated current clinical variables used to determine the risk of death, but none of these variables demonstrated a significant association with increased risk of mortality. In the second model, both clinical variables and gene overexpression were considered, with none of these variables increasing the risk of death. In the third model, MRD and overexpression of selected genes were included, revealing that overexpression of *MIR4435-2HG* emerged as the unique variable that elevated the risk of death 74-fold. Similarly, the fourth model, which evaluated the complete gene profile, indicated that overexpression of *MIR4435-2HG* significantly elevated the risk of death. (Table 3).

**TABLE 3:** Multiple Cox regression using currently clinical variables and gene profile.

Parameter	Model 1. Currently used clinical variables		Model 2. Currently used clinical variables and gene profile		Model 3. Gene profile and MRD		Model 4. Gene profile	
	*p*-value	OR (95%CI)	*p*-value	OR (95%CI)	*p*-value	OR (95%CI)	*p*-value	OR (95%CI)
Age	0.62	NC	0.89	0.20 (0.001–4.79)				
WBC	0.72	NC	>0.99	NC				
Extramedullar infiltration	>0.99	NC	>0.99	NC				
Corticoid response	0.18	NC	>0.99	NC				
MRD + day 15	0.90	NC	0.39	0.14 (0.0002–9.03)	0.29	0.20 (0.002–3.24)		
MRD + end of induction	0.31	NC	0.27	3.71 (0.22–258.3)	0.14	6.82 (0.50–189.3)		
*MIR4435-2HG*			0.17	NC	**0.01**	74.38 (4.18–6502)	**0.007**	22.7 (2.73–327.7)
*DAPK1*			>0.99	NC	0.37	3.12 (0.16–96.95)	0.27	3.23 (0.30–28.07)
*ITGA6*			0.15	0.01 (0.0006–5.48)	0.06	0.03 (0.00002–1.59)	0.11	0.06 (0.001–1.89)
*NPDC1*			>0.99	NC	0.06	13.22 (0.68–631)	0.07	12.62 (1.02–342.6)
*CNKSR3*			0.78	1.53 (0.04–36.46)	0.61	1.17 (0.11–9.55)	0.93	1.09 (0.12–8.72)
*ASCL2*			>0.99	NC	0.60	1.58 (0.08–29.20)	0.99	0.98 (0.05–19.78)
*CTHRC1*			>0.99	NC	0.59	0.43 (0.02–5.49)	0.22	0.25 (0.02–2.42)
*SCL45A3*			>0.99	NC	0.63	2.55 (0.08–45.41)	0.97	1.04 (0.02–72.07)
*BOC*			0.50	0.19 (0.0004–19.70)	0.16		0.51	0.39 (0.02–10.47)

MRD + indicates minimal residual disease positive. NC, No calculable;. WBC, white blood counts. Statistically significant results are shown in bold.

**FIGURE 7:**
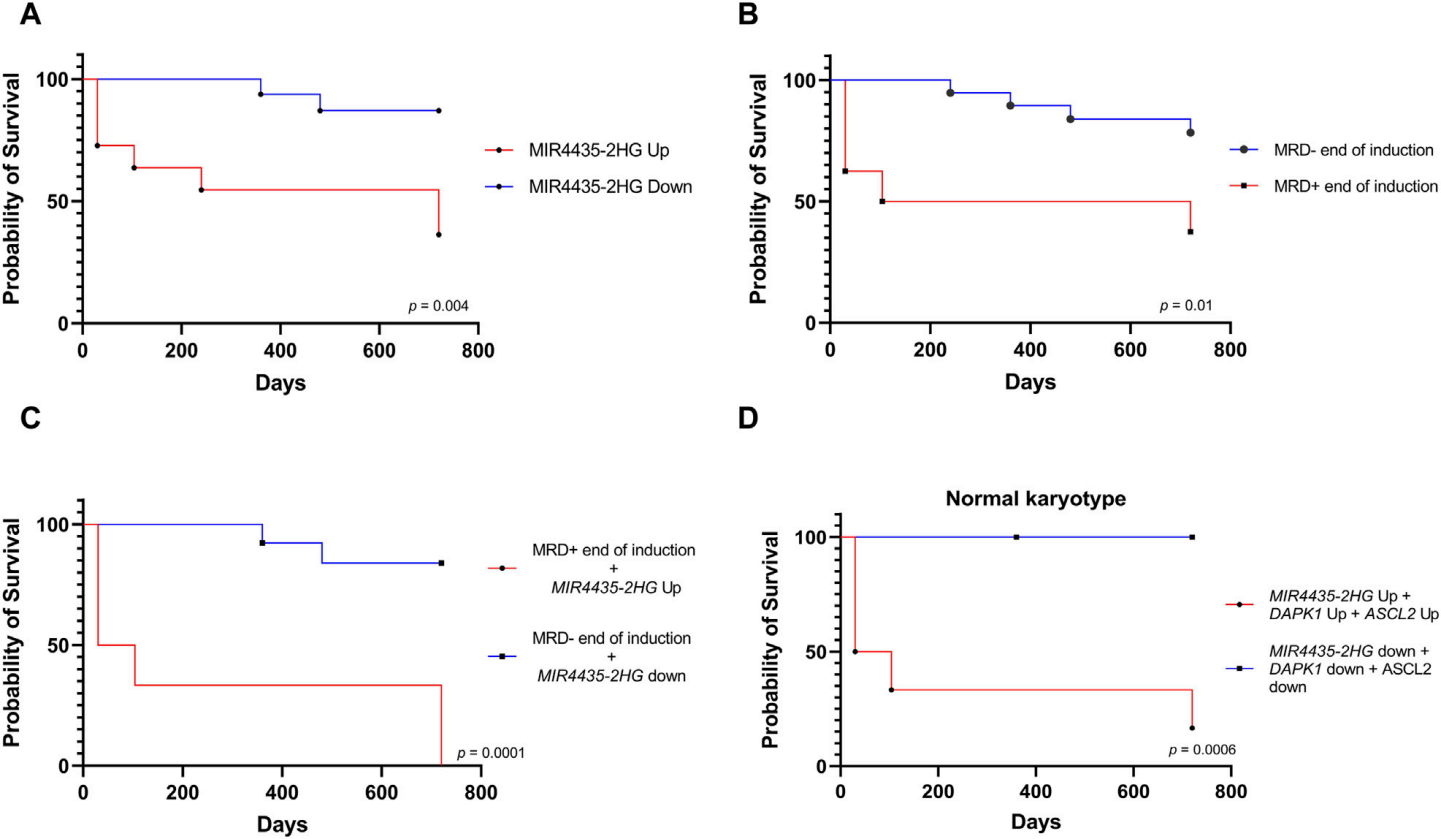
Relationship of the *MIR4435-2HG* expression with death. Kaplan-Meier analysis comparing 2-year survival with respect **(A)** *MIR4435-2HG* expression (*n* = 26); **(B)** MRD—or MRD + at the end of induction treatment (*n* = 26); **(C)** patients with overexpression of *MIR4435-2HG* and MRD + at the end of induction vs patients with low expression of *MIR4435-2HG* and MRD-at the end of induction (*n* = 26); and **(D)** patients with normal karyotype and overexpression or not of *MIR4435-2HG*, *ASCL2*, and *DAPK1* (*n* = 15). *p*-value = <0.05.

In line with the previous result, survival analysis showed that patients with *MIR4435-2HG* overexpression had worse survival; however, it is important to validate this result in a larger cohort of patients (Figure 7A). Since MRD is the current variable most commonly used to define the risk of death, however, for the survival analysis we first considered MRD at the end of induction, effectively demonstrating that MRD-patients have better survival than MRD + patients. (Figure 7B). Importantly, a more accurate separation of survival curves was achieved when we compared the survival of patients combining MRD+ with *MIR4435-2HG* overexpression *versus* MRD-patients with *MIR4435-2HG* down-expression. This revealed that patients with MRD+ and *MIR4435-2HG* overexpression experienced markedly worse survival (Figure 7C).

Finally, given that more than half of our patients had normal karyotype, we evaluated whether selected genes could improve risk classification in this subgroup of patients. Remarkably, the simultaneous overexpression of *MIR4435-2HG*, *DAPK1*, and *ASLC2* was associated with worse survival in patients with normal karyotype compared to those who did not overexpress them (Figure 7D).

## 4 Discussion

Given the wide genetic and epigenetic heterogeneity inherent in ALL, there is a critical need for new biomarkers to improve the prognosis of patients (Lejman et al., 2022). The present study conducted an integrative analysis of genome-wide DNA methylation and gene expression by RNA-seq in a cohort of 14 pediatric patients with B-cell ALL to explore whether differential DNA methylation genes and gene expression patterns could be proposed as potential predictive biomarkers that differentiate responder from non-responder patients and confer risk of death in pediatric patients with B-cell ALL.

Aberrant DNA methylation has been considered a hallmark in different types of cancer, including ALL (McCabe et al., 2009; Hanahan, 2022). Consistent with the findings of Borssén et al., 2018, our study demonstrated a clear separation in both DNA methylation and gene expression profiles between MRD- and MRD + patients. Notably, the overexpression of genes was associated with a more aggressive phenotype. Previously, Figueroa et al., 2013 reported that aberrant DNA methylation in childhood ALL could play a crucial role as a determinant of gene expression in disease-specific alterations. In our study, we observed a negative correlation between hypomethylation of CpGs and overexpression of genes *DAPK1*, *CNKSR3*, *MIR4435-HG2*, *CTHRC1*, *NPDC1*, *SLC45A3*, *ITGA6*, *ASCL2,* and *BOC*, supporting the idea that changes in DNA methylation have the potential to influence gene expression. While it is widely recognized that promoter methylation can influence gene expression (Moore et al., 2013), the specific biological mechanisms driving this alteration in leukemias remain unclear. Some researchers have proposed several approaches that may be linked to mutations in the epigenetic machinery. For instance, mutations in DNMTAs have been associated with a gain of function in the protein, leading to either global or segmented hypermethylation (Schulze et al., 2016; Brunetti et al., 2017). Conversely, alterations in DNA demethylation mechanisms, such as gain-of-function mutations in TET enzymes, can result in zones of hypomethylation (Huang et al., 2013; Bowman and Levine, 2017; Wu and Zhang, 2017). Additionally, some studies have suggested a correlation between altered methylation states and the availability of the substrate SAM (S-adenosylmethionine), indicating that a low dietary intake of SAM-containing foods could impact an individual’s methylation states (Mentch et al., 2015).

However, this study does not fully elucidate the biological mechanism underlying hypomethylation associated with gene overexpression. Nevertheless, these findings provide valuable insights that can help generate new hypotheses to further understand the underlying biological mechanisms.

Aberrant patterns of DNA methylation have been linked to clinical outcome in patients with ALL; however, further research is required to evaluate the clinical utility of some of these findings (Tsellou et al., 2005; Roman-Gomez et al., 2007; Kuang et al., 2008; Musialik et al., 2015; Mai et al., 2016; Ogawa et al., 2016). In this study, we selected *MIR4435-2HG*, *DAPK1*, *ASCL2*, *BOC*, and *CNKSR3* as potential biomarkers of treatment response. Their overexpression reliably predicts treatment failure or refractoriness with high sensitivity and specificity. Notably, among these biomarkers, *MIR4435-2HG* stands out as the most robust predictor of therapeutic failure.

Recently, selected genes had been described as possible diagnostic and prognostic biomarkers in different types of cancer and other non-neoplastic diseases (Table 4).

**TABLE 4:** List of genes choose as possible predictive biomarkers of induction chemotherapy response.

Gene name	FC	*p*-value	Rho	Cancer association	Ref
*DAPK1*	3.9	0,000009	−0.91	Gastric, pancreatic, head and neck, thyroid, brain, uterine, lung, esophageal cancers, CLL, AML and MDS	Calmon et al. (2007), Greco et al. (2010), Qin et al. (2014), Wang et al. (2014), Yuan et al. (2017), Wei et al. (2020), Gasimli et al. (2022), Guru et al. (2022), Movahhed et al. (2022)
*CNKSR3*	5.7	0,0003	−0.85	Melanoma	Lake et al. (2013)
*SLC18A2*	8.7	0,0009	−0.8	Prostate cancer and AML	Sørensen et al. (2009), Lebedev et al. (2019)
*CTHRC1*	5.5	0,003	−0.76	Renal, head and neck, liver, stomach, lung, endometrial and colorectal cancers	Sial et al. (2021), Meng et al. (2022)
*NPDC1*	4.4	0,003	−0.74	Gastric, neuroblastoma, pancreatic neuroendocrine tumors and AML	Bloomston et al. (2004), Tong et al. (2013), Nguyen et al. (2019), Dong et al. (2022)
*BOC*	4.6	0.01	−0.68	Gastric and pancreatic cancers	Mathew et al. (2014), Fattahi et al. (2021)
*ASCL2*	5.4	0,004	−0.66	Colorectal, gastric and lung cancer. Risk of ALL in pregnancy	Kwon et al. (2013), Hu et al. (2016), Potter et al. (2018), Zuo et al. (2018), Wu et al. (2022)
*SLC45A3*	3.9	0.03	−0.59	Prostate cancer	Esgueva et al. (2010)
		0,04	−0.57		
*ITGA6*	3.8	0.02	−0.63	Multiple myeloma and AML	Yamakawa et al. (2012), Song et al. (2021)
MIR4435-2HG	4.4	0.02	−0.55	Digestive, reproductive, respiratory, nervous, and urinary tumors, AML and T-cell ALL	Ouyang et al. (2019), Shen et al. (2020), Zhu et al. (2020), Ghasemian et al. (2022), Zhong et al. (2022)

In particular, *MIR4435-2HG*, which is a long non-coding RNA, is also known as *LncRNA-AWPPH*, *LINC00978*, or *MORRBID* (Ghasemian et al., 2022). Interestingly, *MIR4435-2HG* overexpression has previously been associated with hypomethylation in gliomas (Zhong et al., 2022). In patients with T-cell ALL, *MIR4435-2HG* showed an elevated expression compared to healthy individuals and has been linked to the promotion of proliferation as well as the inhibition of apoptosis of ALL cell lines (Li et al., 2020). Although the precise biological role of *MIR4435-2HG* is still under investigation, it is known to contribute by deregulating different signaling pathways associated with proliferation, invasion, migration, epithelial-mesenchymal transition, and apoptosis. Specifically, it plays a role in signaling pathways such as TGF-β, WNT-β catenin, MDM2/p53, PI3K/AKT, Hippo, and MAPK/ERK (Ouyang et al., 2019; Zhong et al., 2022).

Unfortunately, no clinical variable was identified as risk factor for death in our population; however, *MIR4435-2HG* overexpression was found to significantly increases risk of death, predicts death, and correlated with poorer survival. Similar findings have been reported in acute myeloid leukemia by Zhigang Cai et al., 2020. Moreover, in other cancer models, *MIR4435-2HG* overexpression has consistently been associated with worse progression-free survival and overall survival (Ouyang et al., 2019; Zhu et al., 2020; Zhong et al., 2022).

No studies have explored the relationship between aberrant DNA methylation, gene overexpression, and MRD during induction chemotherapy. However, certain authors have reported differences in the methylation profiles of patients who experienced relapse compared to those who did not (Borssén et al., 2018), where patients with a less methylated CpG island methylator phenotype at diagnosis exhibited inferior overall survival compared to those with more methylated CpG island phenotype. In a prior study, Sandoval et al., 2012 reported hypomethylation in various genome regions, including Polycomb target genes, and its association with poor survival and relapse. Similarly, Hogan et al. reported epigenetic dysregulation in the acquisition of chemoresistance during relapse, involving genes *CDKN2A*, *COL6A2*, *PTPRO*, and *CSMD1* (Hogan et al., 2015).

The search for biomarkers in the transcriptome or methylome of patients is very valuable, especially when 25% of patients with pediatric leukemia lack detectable genetic alterations and have a low mutation rate, which is a challenge for risk classification (Iacobucci and Mullighan, 2017). Interestingly, in our cohort, more than 70% of our patients showed no genetic alterations but displayed overexpressed of *MIR4435-2HG*, *DAPK1* and *ASCL2*, which correlated with worse survival. These results suggest that assessing the expression of these genes by RT-qPCR could improve risk classification, especially in patients without genetic alterations.

While *MIR4435-2HG* overexpression appears to be a poor prognostic factor, the association of *DAPK1* expression with poor prognosis is controversial (San Jose-Eneriz et al., 2013). In this study, we associated *DAPK1* overexpression and hypomethylation with therapeutic failure, and poorer survival in patients with normal karyotype. *DAPK1* has also been associated with resistance to imatinib in chronic myeloid leukemia (Guru et al., 2022), autophagia (Singh et al., 2016), alterations in the p53 signaling pathway in chronic lymphocytic leukemia (Wang et al., 2014) and methylated in myelodysplastic syndrome (Greco et al., 2010).

**FIGURE 8:**
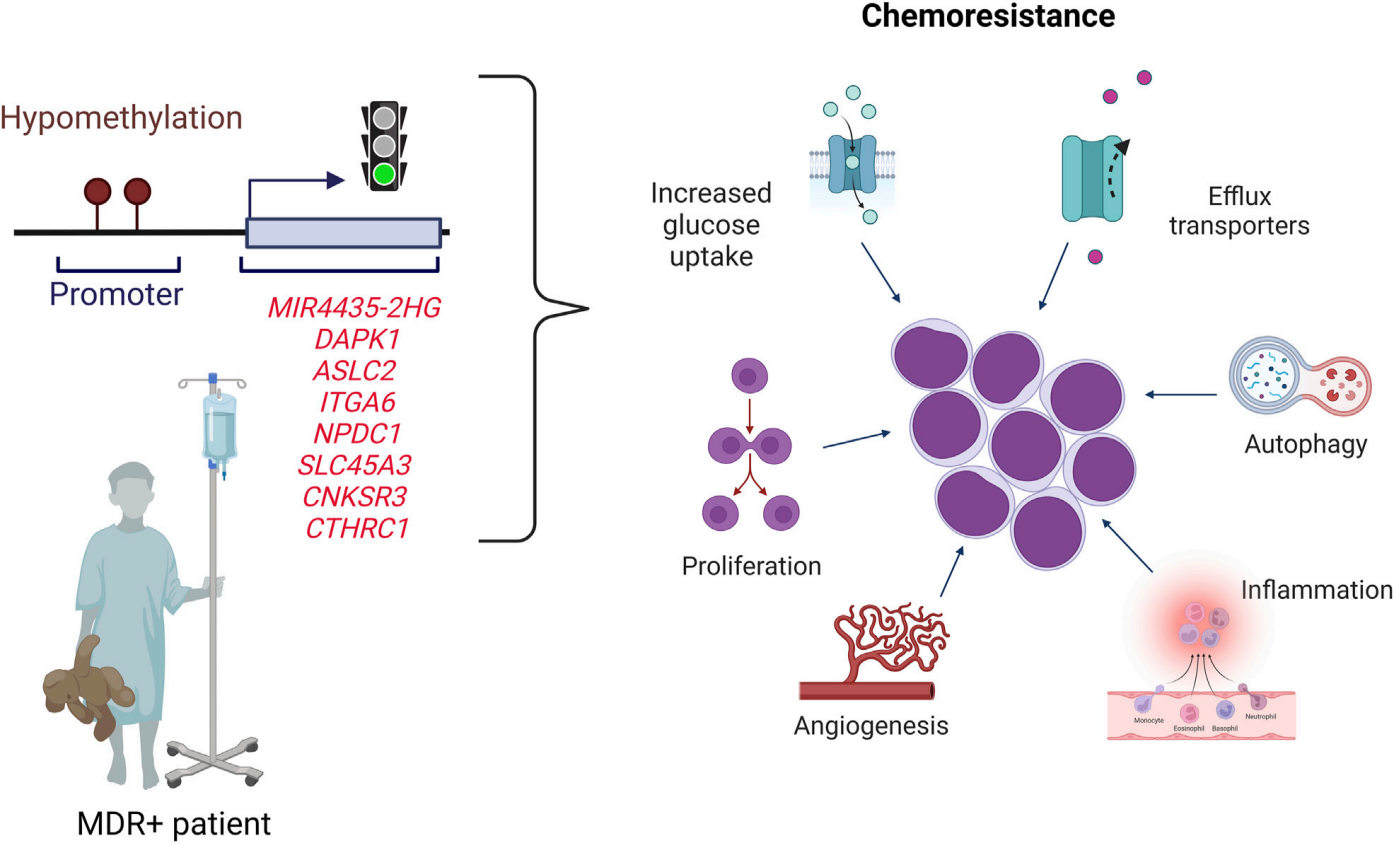
Proposed scheme for pediatric B-cell ALL patients. Patients with B-ALL who overexpress the *MIR4435-2HG*, *DAPK1*, *ASCL2*, *ITGA6*, *NPDC1*, *SLC45A3*, *CNKSR3* and *CTHRC1* genes have a high probability of being MRD+ and dying. The overexpression could be related to hypomethylation of the CpGs sites of these genes. The genes overexpressed are related to different hallmarks of cancer.

Here we propose a gene profile that can predict treatment response in children with B-ALL In particular, we demonstrate for the first time that *MIR4435-2HG* is overexpressed and hypomethylated in MRD + patients, and that it has the ability to predict treatment response and confer an increased risk of death in those patients who overexpress it (Figure 8). The detection of *MIR4435-2HG* could be combined with MRD analysis to improve risk classification, particularly in patients with normal karyotype. The proposed genetic profile offers the possibility of expanding research into new biomarkers predictive of response to treatment, which, in the future, would be a valuable tool to improve risk classification. A great contribution of this study is that the genes can be identified by RT-qPCR, which is efficient, fast, and cost-effective at the clinical level.

The limitations of this study are associated with the relatively low number of newly diagnosed patients eligible for this investigation, as well as the limited number of MRD + patients. Another limitation is associated with the follow-up duration for the patients, typically limited 2 years in most cases, which does not allow us to generate a solid conclusion about patient survival (death). Therefore, it is crucial to validate these results in a larger cohort of patients with a prolonged follow-up period for a more comprehensive evaluation.

## Data Availability

The datasets presented in this study can be found in online repositories. The names of the repository/repositories and accession number(s) can be found below: Gene Expression Omnibus (GEO), accession number GSE229056.
