# Supplementary material for: MIR4435-2HG as a possible novel predictive biomarker of chemotherapy response and death in pediatric B-cell ALL
Source: Front Mol Biosci. 2024 Apr 30;11:1385140. doi: 10.3389/fmolb.2024.1385140 (PMC11091394; doi:10.3389/fmolb.2024.1385140)
Supplement: Supplementary file 2 [file Table1.pdf]

TABLE 1. CLINICAL CHARACTERISTICS OF THE VALIDATION COHORT (n=18)		
**CLINICAL CHARACTERISTICS**	**n (%)**	**MEAN (RANGE)**
**AGE (years)**		8.26 (1-15)
**SEX**		
Female	9 (50)	
Male	9 (50)	
WBC (cel/m)		40.39 (0.8-269)
**RISK**		
Low	3 (16.7)	
Intermediate	11 (61.1)	
High	4 (22.2)	
**EXTRAMEDULLAR INFILTRATION**		
Yes	3 (17)	
No	15 (83)	
**CORTICOID RESPONSE**		
Yes	17 (94)	
No	1 (6)	
**CARIOTYPE/MOLECULAR ALTERATIONS**		
Normal	11 (61.1)	
Hipodiploid	0 (0)	
Hyperdiploid	3 (16.7)	
t(1;19)	1 (5.6)	
t(4;11)	0 (0)	
t(9;22)	0 (0)	
t(12;21)	1 (5.6)	
Amp Cr 21	0 (0)	
Other	2 (11.1)	
**MRD DAY 15**		
Positive	11 (61)	
Negative	7 (39)	
**MRD END OF INDUCTION**		
Positive	3 (17)	
Negative	15 (83)	
**RELAPSE**		
Yes	3 (17)	
No	15 (83)	
**DEATH**		
Yes	3 (17)	
No	15 (83)	
Minimal residual disease (MRD). White blood count (WBC).		
