# Supplementary material for: MIR4435-2HG as a possible novel predictive biomarker of chemotherapy response and death in pediatric B-cell ALL
Source: Front Mol Biosci. 2024 Apr 30;11:1385140. doi: 10.3389/fmolb.2024.1385140 (PMC11091394; doi:10.3389/fmolb.2024.1385140)
Supplement: Supplementary file 3 [file DataSheet1.docx]

**Supplementary material**

**Title:** *MIR4435-2HG* AS A POSSIBLE NOVEL PREDICTIVE BIOMARKER OF CHEMOTHERAPY RESPONSE AND DEATH IN PEDIATRIC B-CELL ALL

**Yulieth Torres-Llanos**, **Jovanny Zabaleta**, **Nataly Cruz-Rodriguez**, **Sandra Quijano**, **Paula Guzman**, **Iliana de los Reyes**, **Nathaly Poveda-Garavito**, **Ana Infante**, **Liliana Lopez**, **Alba Combita**.

**Supplementary material 2. ROC curves and logistic regression of genes as predictors of MRD-/- or MRD+/+ patients.** Logistic regression analysis and Receiver Operating Characteristic (ROC) curves were employed to assess the predictive capacity, sensitivity, and specificity of the genes. In logistic regressions, the Y-axis represents the probability of one of the outcomes, ranging between 0 and 1, where 1 indicates a lack of response to treatment, and 0 indicates a positive response. The X-axis corresponds to RNA-seq counts for each gene. The figures (A-H) display the results for *DAPK1*, *BOC*, *ASCL2*, *CNKSR3*, *NPDC1*, *SCL45A3*, *ITGA6*, and *CTHRC1*, respectively.
